# PD-L1 Expression on Tumor Cells Is Associated With a Poor Outcome in a Cohort of Caucasian Nasopharyngeal Carcinoma Patients

**DOI:** 10.3389/fonc.2019.01334

**Published:** 2019-11-29

**Authors:** Christoph Minichsdorfer, Felicitas Oberndorfer, Christoph Krall, Gabriela Kornek, Leonhard Müllauer, Christina Wagner, Thorsten Fuereder

**Affiliations:** ^1^Department of Internal Medicine I & Comprehensive Cancer Center, Medical University of Vienna, Vienna, Austria; ^2^Department of Pathology, Medical University of Vienna, Vienna, Austria; ^3^Center for Medical Statistics, Informatics and Intelligent Systems, Medical University of Vienna, Vienna, Austria

**Keywords:** nasopharyngeal carcinoma, PD-L1, PD-1, LAG-3, Caucasian population

## Abstract

**Background:** Nasopharyngeal carcinoma (NPC) is endemic in East Asia but rare in the western world. Programmed death ligand 1 (PD-L1) expression on NPC correlates with clinical outcomes. However, data for Caucasian NPC patients are missing. Thus, we performed this retrospective analysis for investigating the potential association of immune checkpoint protein expression with outcome parameters in Caucasian NPC patients.

**Methods:** Fifty-five patients with NPC treated between 1993 and 2018 at the Medical University of Vienna were identified. After the exclusion of Asian patients, data on baseline demographic, tumor stage, overall survival (OS), and disease-free survival (DFS) of 30 patients were analyzed. Their tumor samples were stained and scored (low vs. high) for PD-L1, programmed death receptor 1 (PD-1), lymphocyte activating gene 3 (LAG3), and cluster of differentiation 8 (CD8) antibodies. Statistical analysis was performed with Kaplan-Meier plots and log-rank test. Estimated hazard ratios of dichotomized analysis were calculated, together with 95% confidence intervals and *p*-values of Wald tests.

**Results:** PD-L1 expression was ≥50% in 6 (20%) patients, whereas 19 (63%) had ≥1% expression and 5 (17%) tumor samples were PD-L1-negative. While sex and age had no impact on DFS or OS, <50% PD-L1 expression on tumor cells (TC) was associated with a significantly longer OS (log rank test *p* = 0.037; HR 0.275; 95% CI 0.073–1.03). There was no influence on DFS (log rank test *p* = 0.34; HR 0.599; 95% CI 0.208–1.728). However, <10% PD-L1 expression on tumor infiltrating lymphocytes (TILs) was correlated with a worse DFS (log rank test *p* = 0.0057; HR 4.06; 95% CI 1.389–11.868). LAG3 expression or the number of TILs did not play any prognostic role in our population.

**Conclusion:** The PD-L1 expression rate on Caucasians was comparable to that in Asian patients. Although these results have to be interpreted with caution due to the limited number of Caucasian patients available, our data suggest that ≥50% PD-L1 expression on TC is associated with a poor outcome, while ≥10% PD-L1 expression on TILs is correlated with improved DFS. A prospective biomarker analysis of a predefined Caucasian NPC subpopulation would be desirable in future trials.

## Introduction

The incidence of nasopharyngeal carcinoma (NPC) varies drastically between the Caucasian and Asian populations in Europe/the USA and certain parts of Asia. The incidence in the western world lies between 0.5 and 2 cases per 100,000 people; therefore, NPC is regarded as an orphan disease in these countries. By contrast, NPC is endemic to southern China and Hong Kong with an incidence reaching 25 cases per 100,000 (1–3). In the endemic regions NPC is mainly associated with Epstein Barr virus (EBV) infection, whereas in the USA and Europe, alcohol and tobacco use are the major risk factors for the development of NPC (4, 5). The World Health Organization distinguishes three histological subtypes of this disease. Type I is the keratinizing squamous cell carcinoma, which represents the sporadic form. The non-keratinizing subtypes are further divided into types II and III (differentiated and undifferentiated non-keratinizing carcinoma, respectively). Type III is strongly associated with EBV infection and accounts for almost 95% of NPC in the endemic regions of south-east Asia (6–8). Irrespective of the subtype, treatment for intermediate and locally advanced NPC comprises concurrent chemoradiation preceded or followed by systemic chemotherapy, according to the NCCN guidelines (9, 10). Of note, survival outcomes and response to chemoradiation markedly differ between Asian and non-Asian patients, most likely reflecting the differences underlying tumor biology between the two groups, as demonstrated in a recent population-based analysis (11).

In an attempt to improve the prognosis of locally advanced NPC, intensive research efforts focusing on the evolving landscape of NPC immuno-oncology have been made during the last couple of years. NPC is regarded as a highly immunogenic tumor characterized by high rates of tumor infiltrating lymphocytes (TILs) (12, 13). Furthermore, preclinical investigations suggest that EBV-driven NPC cells up-regulate critical immune checkpoint proteins including programmed death ligand 1 (PD-L1) (14). Clinical data on the potential significance of PD-L1 overexpression in NPC are conflicting; while some studies have proposed a detrimental effect of elevated PD-L1 expression on outcome parameters, others have shown a positive association of PD-L1 expression with survival in NPC patients (15–18). These studies, however, were conducted solely in Asian populations. Neither clinical data nor analyses characterizing the infiltration of TILs and the role of PD-L1, PD-1, or alternative checkpoints proteins such as LAG3 in Caucasian NPC patients that take into account the distinct tumor biology of this population are available so far. The clinical impact of the PD-1/PD-L1 axis and LAG3 on the outcome of Caucasian NPC is currently unclear.

Based on this background, we performed the present retrospective analysis in order to investigate the expression levels of PD-L1 on TC and PD-1 and LAG3 on TILs in a non-Asian NPC patient population and subsequently evaluate the correlation of checkpoint protein expression with overall survival (OS) and disease-free survival (DFS).

## Patients and Methods

### Data Collection

Patients eligible for this single center retrospective analysis had histologically confirmed NPC diagnosed between 1st January 1993 and 30th September 2018 at the Medical University of Vienna. A total of 55 patients were identified. Out of this, histological specimens were available from 37 patients. Two patients were lost to follow up and therefore unavailable for our analysis. An additional five patients were excluded according to the study plan due to their Asian background. Therefore, 30 cases were found to be eligible. All 30 patients received a combination of radiotherapy (R) (66–72 Gray) and chemotherapy (CHT), either as concurrent radiochemotherapy (RCHT), neoadjuvant CHT, or adjuvant CHT. Survival analysis was monitored in September 2018.

The study was performed in accordance with the Declaration of Helsinki and good clinical practice guidelines and was approved by the local ethics committee (EK 1416/2017).

### Tissue Analysis

Samples of histologically proven, formalin fixed, paraffin embedded tissue of nasopharyngeal squamous cell carcinoma were examined retrospectively. For histopathological reports, EBV status was assessed by *in-situ* hybridization (using Ventana EBER Probe 800-2842, Roche Diagnostics GmbH, Mannheim, Germany or Bond EBER Probe PB0589, Leica Biosystems, Nussloch, Germany) using an automated BenchMark Ultra, Roche/Ventana or Leica BOND III, Leica Biosystems immunostainer, respectively. The patients were either biopsied or they underwent surgical treatment at the Medical University of Vienna, Department of Otorhinolaryngology, Head and Neck Surgery. Archived hematoxylin/eosin stained slides were re-evaluated by an experienced pathologist, and the most representative area for each case was chosen. Representative areas were marked and measured and were of dimensions of at least 6 and 25 mm^−2^ for biopsies and for surgical resections, respectively.

### Immunohistochemistry (IHC)

IHC analysis was performed on freshly cut 3 μm thick serial sections of the selected formalin fixed, paraffin embedded tissues. IHC staining was conducted on automated immunostainers (BenchMark Ultra, Roche/Ventana and Leica BOND III, Leica Biosystems) with suitable positive and negative controls. The commercially available antibodies PD-L1 (clone 22C3, mouse monoclonal, Dako, CA, USA, dilution 1:50) and LAG3 (clone 17B4, mouse monoclonal, LifeSpan BioSciences Inc., WA, USA, dilution 1:100) were employed for estimating expression levels of PD-L1 and LAG3, respectively. IHC assays were carried out according to the manufacturer's instructions, after antigen recovery with heat-induced epitope retrieval (HIER) in Cell Conditioning 1 (CC1) buffer (Ventana Medical Systems, AZ, USA) using a standardized in-house protocol. IHC staining of PD1 (clone 315M-96, mouse monoclonal, Cell Marque, CA, USA, dilution 1:50) and CD8 (clone M7103, mouse monoclonal, Dako, CA, USA, dilution 1:100) were conducted on a Leica BOND III automated stainer using a standardized in-house routine protocol with HIER for 20 min with BOND Epitope Retrieval Solution 1 (Leica Biosystems, Nussloch, Germany).

The slides were examined independently by two experienced pathologists and a consensus diagnosis was ascertained. Each IHC staining was analyzed semi-quantitatively in a selected representative area.

Based on the total number of TC in the selected area, the percentage of TC showing specific PD-L1 expression was estimated. Since no validated cut-off values for PD-L1 positivity have been published so far for NPC, we adopted the published cut-offs used in the KEYNOTE-040 study for head and neck squamous cell carcinoma (HNSCC). This was possible, especially since we employed the same antibody clone (i.e., 22C3 from Dako), which was used in the KEYNOTE-040 trial (19). According to these suggested cut-offs, PD-L1-positive membranous staining on a TC proportion of <1% was graded as negative, ≥1– <50% was graded as low, and ≥50% as high at any intensity (19).

IHC expression of PD-L1, PD-1, LAG3, and CD8 on TILs was evaluated as the proportion (percentage) of the tumor infiltrating inflammatory cells.

No validated standards have been determined for PD-L1 positivity on TILs in NPC patients. Therefore, the classification system of the phase II POPLAR trial in lung cancer and the protocol described by Karpathiou et al. in HNSCC were adopted (20, 21) and PD-L1-positive TILs were divided into four groups and three grades (<1% = group 0 = negative, 1–5% = group 1 = low, 5–10% = group 2 = low, and ≥10% = group 3 = high).

No validated cut-off values for LAG-3, PD-1, and CD8 on TILs in NPC samples are established. Therefore, LAG-3, CD8, and PD-1 expression rates for every single patient were determined and the median expression score calculated. Subsequently, all NPC samples were divided into three subgroups according to the aforementioned median of LAG-3 and CD8 immunostaining (adapted as described previously) (22). This scoring method was also employed for PD-1, both for consistency and due to the lack of established PD-1 cut-off values. LAG-3 was graded negative when 0%, low when 1–5%, and high when >5% immunoreactive TILs were detected. Similarly, CD8 was graded negative when 0%, low when 1–35%, and high when >35% immunoreactive lymphocytes were detected. PD-1 expression on TILs was grouped into negative (0%), low (0–1%), and high (>1%).

For survival analysis, the expression of PD-L1 on TC was categorized into a high (≥50%) and a low/negative group (<50%). Additionally, TILs were grouped according to their PD-L1 expression into low/negative (<10%) and high (≥10%) groups. Similarly, we categorized TILs into low/negative (≤1 and ≤5%) and high (>1 and >5%) groups, based on their PD-1 and LAG3 expression.

### Statistical Analysis

To investigate the effects of potential factors for survival, Kaplan-Meier plots for each level were generated and a log rank test was performed. For factors with levels “negative,” “low” and “high,” the analysis was repeated after pooling the classes “negative” and “low” together. Estimated hazard ratios of this dichotomized analysis have been tabled together with 95% confidence intervals and *p*-values of Wald tests. Due to the small number of patients in the study, no control for multiple testing was performed. A two-sided *p*-value <0.05 was considered statistically significant.

## Results

### Patient Characteristics

The demographic and clinical baseline characteristics of the patients involved in this study are depicted in Table 1. The median age of the patients was 56 years (range 22–88 years). Six (20%) patients were female and 24 (80%) male. While only 19 (63%) patients had EBV-positive disease, the predominant histological subtypes were type II (*n* = 12 [40%]) and type III (*n* = 16 [53%]). The distribution of the disease stage, according to the 7th American Joint Committee on Cancer (AJCC) edition, was as follows: I with *n* = 1 (4%), II with *n* = 3 (10%), III with *n* = 13 (43%), and IVa with *n* = 13 (43%).

**Table 1:** Patient characteristics (*n* = 30) (100%).

**Sex**	***n* (%)**
Female	6 (20)
Male	24 (80)
**Age (years)**	
Median	56
Range	22–88
**T-stage**	***n* (%)**
T1	5 (17)
T2	10 (33)
T3	4 (13)
T4	11 (37)
**N-stage**	
N0	5 (17)
N1	2 (7)
N2	19 (63)
N3	4 (13)
**American Joint Committee on Cancer (AJJC) 7 stage**	
I	1 (4)
II	3 (10)
III	13 (43)
IVa	13 (43)
**EBV status**	
Positive	19 (63)
Negative	11 (37)
**WHO type**	***n* (%)**
I	2 (7)
II	12 (40)
III	16 (53)
**DFS (months)**	
Median	36.5
Range	1–174
**OS (months)**	
Median	54
Range	1–184
**Treatment**	***n* (%)**
Induction chemotherapy	7 (23)
Primary radiotherapy	6 (20)
Radiochemotherapy	20 (67)
Adjuvant chemotherapy	6 (20)
Surgery/radiation	3 (10)
**Recurrence of disease**	13 (43)

*Please note that treatment modalities add up to more than 100%, since some patients received multiple types of therapy*.

Induction chemotherapy (ICHT) was recommended for 7 patients (23%), of which 6 (20%) received primary radiation and 1 (3%) experienced a lethal ICHT-related adverse event. Twenty (67%) patients were treated with primary RCHT. Adjuvant chemotherapy following RCHT was administered only in a minority of 6 (20%) patients. Three (10%) patients were treated with multimodality surgery/radiation approaches. Recurrent disease after primary curative therapy was recorded in 13 (43%) patients.

The median DFS and OS were 36.5 months (range 1–174 months) and 54 months (1–184 months), respectively.

**Figure 1:**
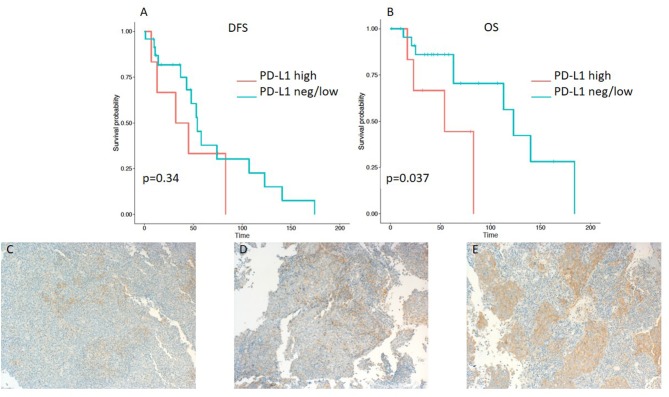
Kaplan–Meier curves for DFS **(A)** and OS **(B)** according to PD-L1 expression on tumor cells (TC) (neg/low vs. high). Representative images of PD-L1 expression on TC: **(C)** negative, **(D)** low, and **(E)** high (x100 magnification). PD-L1, programmed death ligand 1. Log-rank tests were performed, and *p*-values are shown.

**Figure 2:**
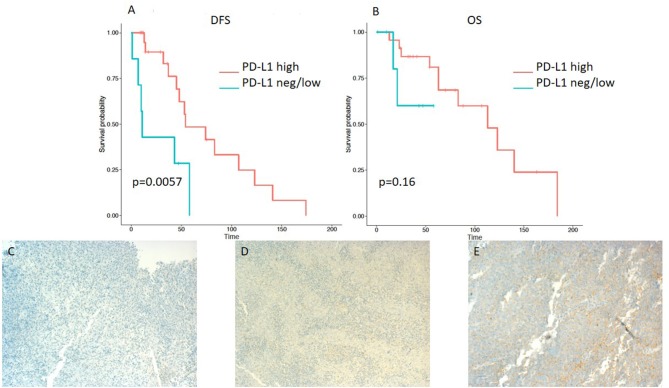
Kaplan–Meier curves for DFS **(A)** and OS **(B)** according to PD-L1 expression on tumor infiltrating lymphocytes (TILs) (neg/low vs. high). Representative images of PD-L1 expression on TILs: **(C)** negative, **(D)** low, and **(E)** high (x100 magnification). Log-rank tests were performed, and *p*-values are depicted.

**Figure 3:**
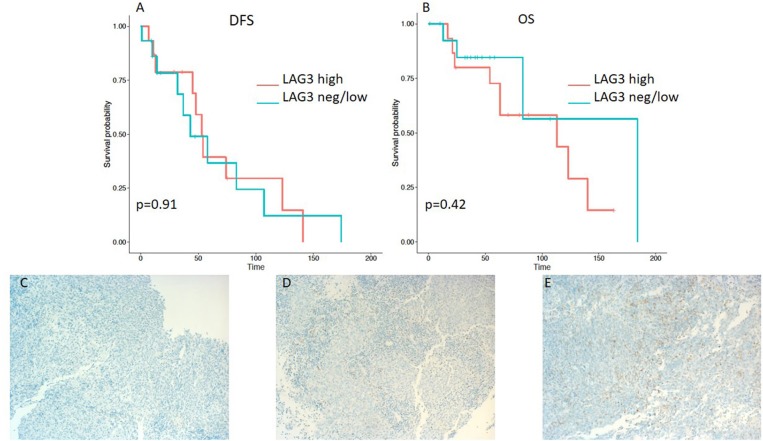
Kaplan–Meier curves for DFS **(A)** and OS **(B)** according to LAG3 expression on tumor infiltrating lymphocytes (TILs) (neg/low vs. high). Representative images of LAG3 expression on TILs: **(C)** negative, **(D)** low, and **(E)** high (x100 magnification). Log-rank tests were performed, and *p*-values are depicted.

**Figure 4:**
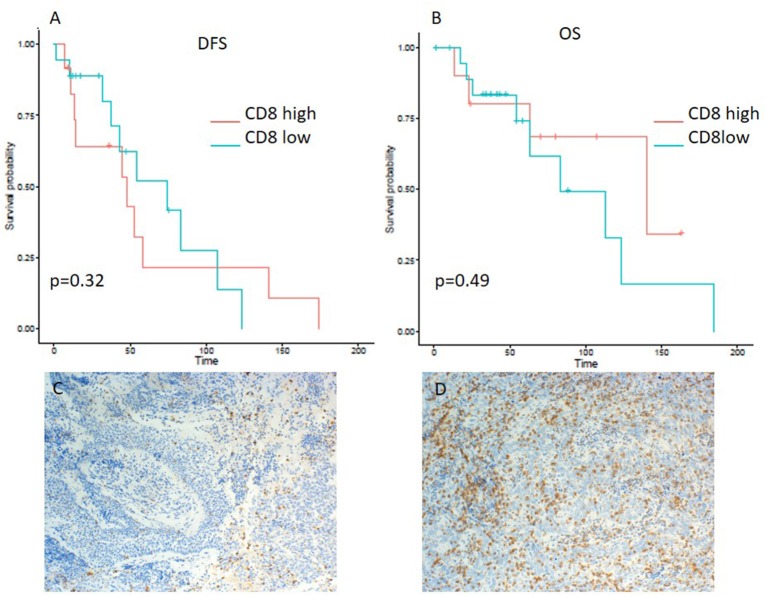
Kaplan–Meier curves for DFS **(A)** and OS **(B)** according to CD8 expression on tumor infiltrating lymphocytes (TILs) (low vs. high). Representative images of CD8 expression on TILs: **(C)** low and **(D)** high (x100 magnification). Log-rank tests were performed, and *p*-values are depicted.

**Figure 5:**
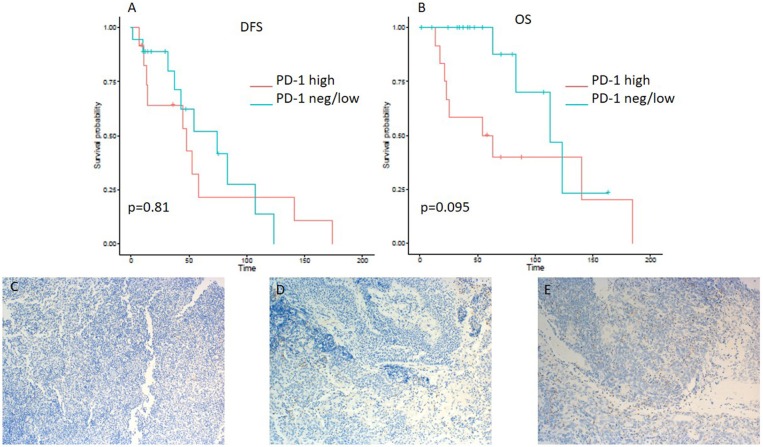
Kaplan–Meier curves for DFS **(A)** and OS **(B)** according to PD-1 expression on tumor infiltrating lymphocytes (TILs) (neg/low vs. high). Representative images of PD-1 expression on TILs: **(C)** negative, **(D)** low, and **(E)** high (x100 magnification). Log-rank tests were performed, and *p*-values are depicted.

A minority of 6 (20%) patients had tumors with high PD-L1 expression, 19 (63%) had a low expression, and 5 (17%) tumor samples were PD-L1-negative (Table 2). PD-L1 on TILs was highly positive in 23 (77%) patients, low in 6 (20%) cases, and negative in 1 (3%) sample. PD-1 expression on TILs was high in 12 (40%) NPC patients, low in 9 (30%) samples, and negative in 9 (30%) cases. CD8-positive TILs were high in 10 (33%) and low in 20 (67%) cases. TILs expressing high levels, low levels, and no LAG3 were detected in 15 (50%), 13 (43%), and 2 (7%) patients, respectively (Table 2). Representative examples of PD-L1, PD-1, LAG3, and CD8 expressing TC or TILs are depicted in Figures 1C–E, 2C–E, 3C–E, 4C,D, 5C–E.

**Table 2:** Immunohistochemical characterization of NPCs studied here.

**PD-L1 expression on tumors**	***n* (%)**
High (≥50%)	6 (20)
Low (≥1 to <50%)	19 (63)
Negative (<1%)	5 (17)
**PD-L1 expression on TILs**	
High (≥10%)	23 (77)
Low (≥1 to <10%)	6 (20)
Negative (<1%)	1 (3)
**Lag3 expression**	
High (>5%)	15 (50)
Low (≥1 to ≤5%)	13 (43)
Negative (<1%)	2 (7)
**PD-1-positive TILs**	
High (>1%)	12 (40)
Low (0 to 1%)	9 (30)
Negative (0%)	9 (30)
**CD8-positive TILs**	
High (<35%)	10 (33)
Low (1 to 35%)	20 (67)
Negative (0%)	0 (0)

### Association Between Checkpoint Inhibitor Protein Expression Level and Survival

Patients with ≤50% PD-L1 expression on tumor samples had a significantly longer OS in the Kaplan-Meier log-rank test analysis (*p* = 0.037), although the hazard ratio for OS did not reach a significant level (HR 0.275; 95% CI 0.073–1.03; *p* = 0.055) (Table 3). Interestingly, there was no difference in DFS (HR 0.599; 95% CI 0.208–1.728; *p* = 0.343 and log-rank test: *p* = 0.34) (Table 3 and Figures 1A,B).

**Table 3:** Association of DFS and OS with clinical and pathological characteristics.

	**HR (95% CI)**	***p*-value**
**DFS**		
Gender	2.601 (0.882–7.672)	0.083
T-stage (T1/2 vs. T3/4	1.536 (0.614–3.841)	0.359
N-stage (N0/1 vs. N2/3)	0.436 (0.15–1.266)	0.127
EBV (pos vs. neg)	0.994 (0.388–2.548)	0.99
PD-L1 tumor (neg/low vs. high)	0.599 (0.208–1.728)	0.343
PD-L1 IC (neg/low vs. high)	4.06 (1.389–11.868)	**0.01**
LAG3 (neg/low vs. high)	1.055 (0.426–2.615)	0.907
CD8 grading (high vs. low)	1.672 (0.598–4.674)	0.327
PD-1 (neg/low vs. high)	0.889 (0.347–2.277)	0.806
**OS**		
Gender	1.668 (0.43–6.476)	0.46
T-stage (T1/2 vs. T3/4	2.292 (0.676–7.772)	0.183
N-stage (N0/1 vs. N2/3)	0.319 (0.079–1.285)	0.108
EBV (pos vs. neg)	1.99 (0.524–7.553)	0.312
PD-L1 tumor (neg/low vs. high)	0.275 (0.073–1.03)	0.055
PD-L1 IC (neg/low vs. high)	3.192 (0.57–17.895)	0.187
LAG3 (neg/low vs. high)	0.577 (0.152–2.191)	0.419
CD8 grading (high vs. low)	1.538 (0.451–5.248)	0.492
PD-1 (neg/low vs. high)	0.356 (0.106–1.197)	0.095

*Estimated hazard ratios of this dichotomized analysis are tabled together with 95% confidence intervals and p-values of Wald tests. Bold value indicates a significant difference*.

On the other hand, lower expression of PD-L1 on TILs (<10%) was associated with a shorter DFS, as evident from a log-rank test (*p* = 0.0057). Additionally, a significant difference in hazard ratios between these two classes was identified employing a Wald test in a Cox proportional-hazards model (HR 4.06; 95% CI 1.389–11.868; *p* = 0.01) (Table 3). In contrast to the OS impact of PD-L1 expression on TC, no difference in OS was detected for PD-L1 expression on TILs (Figures 2A,B). Likewise, PD-1 expression on TILs had no significant influence on OS (HR 0.356; 95% CI 0.106–1.197; *p* = 0.095) or on DFS (HR 0.889; 95% CI 0.347–2.277; *p* = 0.806), as depicted in Table 3. Apart from that, no difference in DFS or OS for negative/low and high LAG3 expressing patients was detected (HR 1.055; 95% CI 0.426–2.615; *p* = 0.907; log-rank test: *p* = 0.91; and HR 0.577; 95% CI 0.152–2.191; *p* = 0.419; log-rank test: *p* = 0.42. respectively) (Table 2 and Figures 3A,B). We further analyzed potential associations of CD8 expression and PD-1 on TILs with survival. However, none of these factors had a significant influence on OS (HR 1.538; 95% CI 0.451–5.248; *p* = 0.49 for CD8 and HR 0.356; 95% CI 0.106–1.197; *p* = 0.095 for PD-1) or DFS (HR 1.672; 95% CI 0.598–4.674; *p* = 0.327 for CD8 and HR 0.889; 95% CI 0.347–2.277; *p* = 0.806 for PD-1) (Table 3 and Figures 4A,B, 5A,B).

## Discussion

In this retrospective study, we highlight a potential association between tumor PD-L1 expression on TC and decreased OS in a Caucasian cohort of NPC patients. Our study shows that, surprisingly, a higher rate of PD-L1-positive TILs is correlated with a longer DFS.

The presently available literature investigating the expression of PD-L1 on both TC and TILs in NPC patients focuses solely on patients in endemic areas and, therefore, these reports uniformly come from Asian cohorts.

Ma et al. reported that ≥1% PD-L1 expression was present in 40% TC and in 22.2% TILs, while no effect on OS or DFS was shown (23). Such a low expression rate of PD-L1 has also been observed in some retrospective studies, with levels of 20–40% (18, 24–26). On the other hand, several retrospective studies have reported PD-L1 positivity and high PD-L1 expression rates in up to 70–100 and 34–46% of NPC tumor samples, respectively (14, 16, 17, 27, 28). In our analysis, the PD-L1 positivity rate was 83%, which is in line with existing literature and similar to that in an Asian population.

There could be multiple additional explanations for the discrepancies regarding PD-L1 expression reported in the literature. Notably, no uniform antibody was used for IHC staining for PD-L1 and staining protocols differed markedly in the above-mentioned studies. In fact, a recent work highlighted that PD-L1 IHC staining results differ strongly depending on the antibody utilized (29). Apart from that, no validated PD-L1 scoring algorithm for NPC has so far been developed and there is no widely accepted consensus regarding the cut off values for PD-L1 positivity. Of note, we used the PD-L1 (clone 22C3) mouse monoclonal antibody from Dako, which is included in the FDA approved diagnostic tool for PD-L1 staining in various solid malignancies.

The expression of PD-L1 in NPC is upregulated by EBV-induced latent membrane protein 1 (LMP1): In an *in vitro* study, EBV-negative cell lines showed lower PD-L1 expression than EBV-positive cell lines (14). According to literature, 90% of endemic NPCs in southeast Asia are EBV-associated (30). In our Caucasian cohort, only 63% of the patients showed EBV association, and in line with this observation, only a minority of the tumors (20%) showed high PD-L1 expression on tumor cells. This probably reflects the high proportion of EBV-negative patients, who harbor a markedly different immunological tumor microenvironment from EBV-positive patients (31).

Multiple conflicting reports regarding the prognostic role of PD-L1 expression on NPC TC have been published for Asian patients, although the interpretation of these results has to be performed with caution, since different antibodies, staining techniques, and inclusion criteria were utilized. Zheng et al. proposed that a higher PD-L1 expression on TC translates into worse OS (28). In contrast, two other studies did not report a correlation of survival with PD-L1 expression on TC of NPC patients (3, 26) and one study even proposed an improvement of OS with higher PD-L1 expression (18). Additionally, in a cohort of 132 Asian NPC patients, higher PD-L1 expression translated into a worse prognosis in terms of DFS. Furthermore, the distant metastasis free survival was poorer in patients with high PD-L1 expression, as shown in a study of 99 NPC patients (24) (overview summarized in Table 4). Very recently, a meta-analysis on the prognostic significance of PD-L1/PD-1 in NPC patients has been published (32). This study shows that the PD-L1/PD-1 detection methods and cut-off values differed markedly between the analyzed reports and no correlation of PD-L1/PD-1 expression with survival in Asian NPC patients was demonstrated (32).

**Table 4:** Studies investigating the association of outcome parameters and PD-L1 expression in NPC.

**References**	***n***	**PD-L1 antibody**	**Cells**	**Cut-off values**	***n* per group**	**Outcome**
Zhou et al. (16)	115	E1L3N, cell signaling	Tumor	H-score < 115, ≥115	38, 61	Worse OS
			TILs	–	–	–
Zhang et al. (17)	160	E1L3N, cell signaling	Tumor	H-score ≤ 35, >35	69, 58	Worse DFS
			TILs	–	–	–
Lee et al. (18)	160	E1L3N, cell signaling	Tumor	<5, 5–24, ≥25%	78, 4, 22	Better LRFS, PFS
			TILs	–	–	–
Qu et al. (24)	96	ab205921, abcam	Tumor	≤10, >10%	68, 28	Worse DMFS
			TILs	–	–	–
Chan et al. (26)	161	SP142, Ventana	Tumor	<1, 1–4, ≥5%	122, 39, 24	No correlation with PFS, OS
			TILs	<1, 1–4, ≥5%	40, 121, 38	No correlation with PFS, OS
Zheng et al. (28)	116	E1L3N, cell signaling	Tumor	0–50, >50%	87, 29	Worse OS
			TILs	–	–	–
Ooft et al. (31)	92	ab58810, abcam	Tumor	–	–	–
			TILs	0–45, >45%	30, 43	Better OS, DFS
Ono et al. (3)	66	D3, cell signaling	Tumor	0–4, ≥5%	13, 53	No correlation with PFS, OS
			TILs	0–4, ≥5%	16, 50	No correlation with PFS, OS

*n, Total number of patients; LRFS, local recurrent free survival; PFS, progression free survival; OS, overall survival; DMFS, distant metastases free survival*.

However, another recent study from the Netherlands, which presumably included a high proportion of Caucasian patients in addition to Asian patients, was not included in the aforementioned meta-analyses, but probably better reflects our patient cohort. This study showed a strong association between a better DFS with a higher rate of CD8 infiltration of TILs and PD-L1-positive TILs, which confirms our findings (31). Other potential immune biomarkers such as PD-1 expression on TILs did not have a significant impact on the survival parameters, which is in accordance with existing literature (16). The role of LAG3 expression on TC in NPC remains elusive since we are not aware of any reports evaluating the role of LAG3 in Asian patients and did not find its association with OS and DFS in our Caucasian cohort study.

In conclusion, NPC is an orphan disease in the western world and the vast majority of molecular insights and clinical data on this disease originate from the endemic regions in southeast Asia. Given the limitations of small sample size and the retrospective nature of our analysis, we found that PD-L1 expression on TC and TILs in a Caucasian population is associated with OS and DFS, respectively. A prospective biomarker analysis of a predefined Caucasian NPC subpopulation would be desirable in future trials.

## Data Availability Statement

All datasets generated for this study are included in the article/supplementary material.

## Ethics Statement

The studies involving human participants were reviewed and approved by Ethics committee of the Medical University of Vienna (EK 1416/2017). Written informed consent for participation was not required for this study in accordance with the national legislation and the institutional requirements.

## Author Contributions

Study design was planned and preparation and editing of the manuscript was done by CM, TF, and FO. Data acquisition was performed by TF, CM, FO, CW, and GK. Quality control of data algorithms and data analysis was performed by TF, CM, FO, LM, and CK. The statistical analysis was done by CK. Review of the manuscript was by TF, CM, FO, LM, CW, GK, and CK.

### Conflict of Interest

TF received travel grants from: MSD, BMS, Merck, and Roche. TF is in advisory boards for: MSD, Merck, Astra Zeneca, Roche, and BI; and received honoraria from: MSD, BMS, Roche, Accord, Sanofi, BI, Novartis, and Astra Zeneca. CM received travel grants from: Roche, BMS, MSD, and Merck; and honoraria from BI and Merck. The remaining authors declare that the research was conducted in the absence of any commercial or financial relationships that could be construed as a potential conflict of interest.
